# Molecular evidence that rough endoplasmic reticulum is the site of calreticulin translation in *Petunia* pollen tubes growing in vitro

**DOI:** 10.1007/s00299-015-1777-x

**Published:** 2015-03-03

**Authors:** Anna Suwińska, Robert Lenartowski, Dariusz Jan Smoliński, Marta Lenartowska

**Affiliations:** 1 Laboratory of Developmental Biology, Faculty of Biology and Environment Protection, Nicolaus Copernicus University, Toruń, Poland; 2 Laboratory of Isotope and Instrumental Analysis, Faculty of Biology and Environment Protection, Nicolaus Copernicus University, Toruń, Poland; 3 Department of Cell Biology, Faculty of Biology and Environment Protection, Nicolaus Copernicus University, Toruń, Poland

**Keywords:** Calcium homeostasis, Germinating pollen, Growing pollen tube, Protein synthesis

## Abstract

*****Key message***:**

**In germinating pollen grains and growing pollen tubes, CRT is translated on ER membrane-bound ribosomes in the regions where its activity is required for stabilization of tip-focused Ca** ^**2+**^ **gradient.**

**Abstract:**

Pollen tube growth requires coordination of signaling, exocytosis, and actin cytoskeletal organization. Many of these processes are thought to be controlled by finely tuned regulation of cytoplasmic Ca^2+^ in discrete regions of the tube cytoplasm. Most notably, a mechanism must function to maintain a steep gradient of Ca^2+^ that exists at the tip of growing pollen tube. Several pieces of evidence point to calreticulin (CRT) as a key Ca^2+^-binding/-buffering protein involved in pollen germination and pollen tube growth. We previously hypothesized that in germinating pollen and growing tubes, CRT is translated on the ribosomes associated with endoplasmic reticulum (ER) in the regions where its activity might be required. In this report, we have addressed this idea by identifying the sites where CRT mRNA, CRT protein, *18S* rRNA, and rough ER are localized in *Petunia* pollen tubes. We observed all four components in the germinal aperture of pollen grains and in subapical regions of elongating tubes. These results seem to support our idea that CRT is translated on ER membrane-bound ribosomes during pollen germination and pollen tube growth. In elongated pollen tubes, we found CRT mainly localized in the subapical zone, where ER and Golgi stacks are abundant. In eukaryotic cells, these organelles serve as mobile intracellular stores of easily releasable Ca^2+^, which can be buffered by proteins such as CRT. Therefore, we postulate that subapical-localized CRT is involved in pollen tube growth by maintaining the stable tip-focused Ca^2+^ gradient and thus modulating local Ca^2+^ concentration within the tube cytoplasm.

## Introduction

The angiosperm pollen tube is a highly specialized vegetative cell that is essential for sexual reproduction. The tube contains within its cytoplasm two immotile sperm cells that are delivered to the embryo sac for double fertilization. On a receptive stigma, the pollen tube develops from the germinal aperture of a pollen grain and extends rapidly through the pistil transmitting tract via polarized tip growth. The female tissues provide specific pollen tube-attracting signals that guide the tube to the ovule (see reviews by Boavida et al. 2005; Takeuchi and Higashiyama 2011). However, pollen tubes can also grow in vitro in the absence of pistil guidance mechanisms, implying that tube growth is self-organizing when tip reorientation is not required. Cultured pollen grains and tubes can be easily manipulated and investigated as whole cells, thus allowing detailed study of the cellular processes and regulatory mechanisms involved in polar growth of this specialized plant cell.

The fine structure of angiosperm pollen tubes has been described in several model plants including *Lilium*, *Nicotiana*, and *Petunia*. The polarized cytoplasm of growing pollen tube is divided into four structurally and functionally distinct zones (see reviews by Boavida et al. 2005; Cheung and Wu 2007; Moscatelli and Idilli 2009; Zonia and Munnik 2008; Guan et al. 2013; Hepler et al. 2013): (1) an extreme organelle-free apex, called the clear zone, which contains an inverted cone of Golgi-derived secretory vesicles that fuse with the growing tip to provide cell wall and membrane extensions; (2) a subapical transition region enriched in metabolically active organelles, such as mitochondria, ER, and dictyosomes; (3) a nuclear domain that contains most of the large organelles such as amyloplasts, smaller vacuoles, and the male germ unit (MGU), which consists of two sperm cells and the pollen tube cell vegetative nucleus; and (4) a vacuolar zone where a central vacuole enlarges during tube elongation. Callose deposition at the cell wall isolates the vacuolar domain from the subapical cytoplasmic regions.

Growing angiosperm pollen tubes have highly active and actin-dependent cytoplasmic streaming (see reviews by Ren and Xiang 2007; Cai and Cresti 2009; Staiger et al. 2010; Guan et al. 2013; Hepler et al. 2013). This occurs via long actin cables that extend along the tube shank to the base of the clear zone, where shorter actin bundles form a cortical actin fringe. Actin cables function as a track for translocation (presumably via myosin XI) of MGU, organelles, and vesicles to the apical cytoplasm. As a result, a bidirectional “reverse fountain” cytoplasmic streaming pattern occurs in which cytoplasm flows toward the tip along the edge of the tube and toward the base through the center of the cell. At the base of the clear zone, the cytoplasmic stream reverses direction and vesicles are captured by the dynamic actin fringe. This structure, together with a network of fine microfilaments at the extreme apex, provides actin tracks for membrane-targeted vesicular trafficking to the apical dome.

Pollen tube growth thus requires the coordination of several processes: formation and maintenance of a zoned cytoplasm, regulation of the cytoskeleton via actin-binding proteins, cytoplasmic streaming, cell wall biogenesis, membrane trafficking, and signaling. Extensive studies have revealed that most, if not all, of these processes are regulated by spatial and temporal variations in the level of cytoplasmic Ca^2+^ in distinct pollen tube zones (see reviews by Holdaway-Clarke and Hepler 2003; Hepler et al. 2011, 2013; Konrad et al. 2011; Guan et al. 2013; Steinhorst and Kudla 2013). Pollen tubes display a tip-focused gradient of free Ca^2+^ that starts to form after pollen germination and oscillates at the extreme tip of the pollen tube during its growth (Brewbaker and Kwack 1963; Jaffe et al. 1975; Rathore et al. 1991; Holdaway-Clarke et al. 1997; Iwano et al. 2004, 2009). This gradient requires extracellular Ca^2+^ influx at the tube apex and is very steep at the tip and behind the apical region. Therefore, a growing pollen tube must possess a highly efficient mechanism for reducing the cytosolic Ca^2+^ level in the sub-apex. Several studies suggest that abundant ER in the subapical zone is responsible for uptake of Ca^2+^. For example, the ER lumen in *Arabidopsis* pollen tubes sequesters high levels of Ca^2+^ (Iwano et al. 2009), and both auto inhibited Ca^2+^ ATPases and ER-type Ca^2+^ ATPases are present in pollen (see review by Sze et al. 2006). Additionally, studies in *Petunia* (Lenartowska et al. 2002; Lenartowski et al. 2015), *Nicotiana*(Nardi et al. 2006), and *Haemanthus* (Lenartowska et al. 2009) indicate that the ER of pollen tubes contains CRT, which is a prominent Ca^2+^ buffer in eukaryotic cells (see review by Jia et al. 2009; Michalak et al. 2009).

A few studies indicate that plant CRT is involved in pollen grain formation and pollen tube growth. This protein was found in mature pollen of *Liriodendron* (Navazio et al. 1998) and *Ginkgo* (Nardi et al. 1998). Additionally, high levels of CRT and its transcripts were detected in developing tobacco anthers, especially in pollen tetrads and in the active tapetum, as well as in dry, hydrated, and germinating pollen and growing tubes (Nardi et al. 2006). Finally, we observed strong CRT expression in germinated pollen and elongated tubes of *Haemanthus* (Lenartowska et al. 2009) and *Petunia* (Lenartowska et al. 2001, 2002; Lenartowski et al. 2014, 2015). On the basis of our findings, we speculated that CRT is translated on ER-associated ribosomes in germinating pollen and growing tubes. However, we were unable to carefully test this hypothesis until our recent cloning of the *Petunia hybrida CRT*gene (*PhCRT*; Lenartowski et al. 2014). Here, we have examined localization of *PhCRT* mRNA, CRT protein, *18S* rRNA, and RER in *Petunia* germinating pollen and growing pollen tubes in vitro. Our data provide the first evidence that CRT is translated on the ER membrane-bound ribosomes during pollen tube growth.

## Materials and methods

### Plant material

Freshly collected mature pollen of *Petunia hybrida* (commercial cultivars grown at room temperature) was germinated in liquid culture media containing 0.2 % sucrose, 0.05 % Ca(NO_3_)_2_, 0.01 % MgSO_4_, 0.01 % H_3_BO_4_, 0.01 % KNO_3_, supplemented with 15 % polyethylene glycol 4000(PEG 4000), and 0.4 % 2-(*N*-morpholino)ethanesulfonic acid (MES), and adjusted to pH 6.0. Cultures were incubated at 30 °C for about 2 h, and germination rates and pollen tube lengths were determined by light microscopy every 30 min. Germinating pollen grains and growing pollen tubes (short or elongated tubes) were then prepared for in situ hybridization, immuno labeling, and electron microscopy as described below. The same developmental stages of cultured pollen and pollen tubes were analyzed by applied methods. For immunoblot detection, anthers (dissected from flowers just before anthesis) and dry/germinated pollen were used. All experiments were repeated at least three times during several growing seasons with similar results.

### Fluorescent in situ hybridization (FISH) of *PhCRT* mRNA and *Petunia 18S* rRNA

In vitro cultivated pollen and pollen tubes were fixed with freshly prepared 4 % formaldehyde (Polysciences) in phosphate-buffered saline (PBS), pH 7.2, for 2 h at room temperature followed by 2 hat 4 °C. After fixation, samples were washed twice in PBS and then in 0.01 M citrate buffer, pH 4.8. Fixed pollen grains and tubes were enzymatically digested in a mixture of 1 % cellulase R10 (Serva) and 27 U/ml pectinase (Sigma) in 0.01 M citrate buffer for 15 min at 37 °C. Then, cells were permeabilized for 10 min in 0.1 % saponin (Sigma) and 3 min in 0.1 % Triton X-100 (Sigma) at room temperature (both detergents were prepared in PBS). For FISH detection of *PhCRT* mRNA, a DIG-labeled antisense RNA molecular probe was used (Lenartowski et al. 2014), whereas *Petunia 18S* rRNA was detected using two DNA oligonucleotide probes (CTATAATGTTATCCCATGCTAATGTATACAGAG and TTTAACTGCAACAACTTAAATATACGCTA) complementary to the *Petunia 18S* rRNA sequence (AJ236020) deposited in GeneBank. Both oligonucleotides were labeled at the 5′ end with biotin (Genomed). Additionally, biotin-11-dUTP (Roche) and ChromaTide Alexa Fluor 488-5-dUTP nucleotides (Invitrogen) were added to the 3′ ends with terminal deoxynucleotidyl transferase (Roche). Hybridization signals were visualized by anti-DIG-rhodamine antibody (Roche) or anti-biotin-FITC antibody (Sigma). Pre-hybridization and hybridization were carried out in 50 % formamide, 4 × SSC, 5 × Denhardt’s, 1 mM EDTA, and 50 mM sodium phosphate buffer, pH 7.0, for 30 min at room temperature and then overnight at 50 °C. Negative controls were processed in the same way except that no probe was added. In the final step, DNA was stained with 2 µg/ml 4′, 6-diamidino-2-phenylindole (DAPI, Fluka). Samples were finally placed onto microscope slides covered with Biobond (BBI Solutions), and images were acquired using the software package EZ 2000 Viewer connected to a fluorescence inverted Nikon confocal microscope (PCM 2000-Eclipse TE 300).

### Immunofluorescence localization of CRT protein

In vitro germinating pollen and growing pollen tubes were fixed with freshly prepared 4 % formaldehyde (Polysciences) in 0.1 M PBS, pH 7.2, for 30 min at room temperature. Samples were then washed in PBS, permeabilized with 0.1 % Triton X-100 (Sigma) in the same buffer for 10 min, and then blocked with 3 % BSA in PBS for 15 min at room temperature. After blocking, cells were incubated with a primary polyclonal antibody against maize CRT (CRT PAb, Napier et al. 1995) diluted 1:50 in PBS with 1 % BSA for 1 h at room temperature followed by overnight at 4 °C. Signals were detected using goat anti-rabbit IgG Cy3^®^ secondary antibody (Sigma) diluted 1:100 in PBS with 1 % BSA for 2 h at room temperature. Controls were performed without the primary antibody. In the final step, DNA was stained with 2 µg/ml DAPI. Samples were placed onto microscope slides covered with Biobond, and images were acquired as described above.

The specificity of the CRT PAbin *Petunia* mature anthers, dry pollen grains, and pollen grains germinated in culture medium was verified by Western blot analysis as previously described (Lenartowski et al. 2015). Maize anthers were used as a positive control.

### Transmission electron microscopy of in vitro germinated pollen and elongated tubes

In vitro cultivated pollen and pollen tubes were fixed with 2 %glutaraldehyde in 0.1 M PBS, pH 7.2, for 2 h at room temperature followed by overnight at 4 °C. Then, fixed cells were stuck to small pieces of Thermanox^®^ plastic sheet (Ted Pella) coated with 0.1 % Poly-l-lysine (Sigma). Cells were washed in PBS, post-fixed with 2 % osmium tetroxide (Sigma) for 30 min at room temperature, rinsed with PBS and Milli-Q-filtered water, dehydrated in graduated ethanol concentrations, and then embedded in Poly/Bed^®^ 812 resin (Polysciences) according to the standard protocol. Ultrathin longitudinal sections of germinated pollen grains and elongated pollen tubes were cut on a Leica UTC ultramicrotome equipped with a diamond knife. The sections were collected on copper grids, stained with 5 % uranyl acetate and 0.4 % lead citrate solutions, and examined on a Joel EM 1010 transmission electron microscope.

## Results

**Fig. 1:**
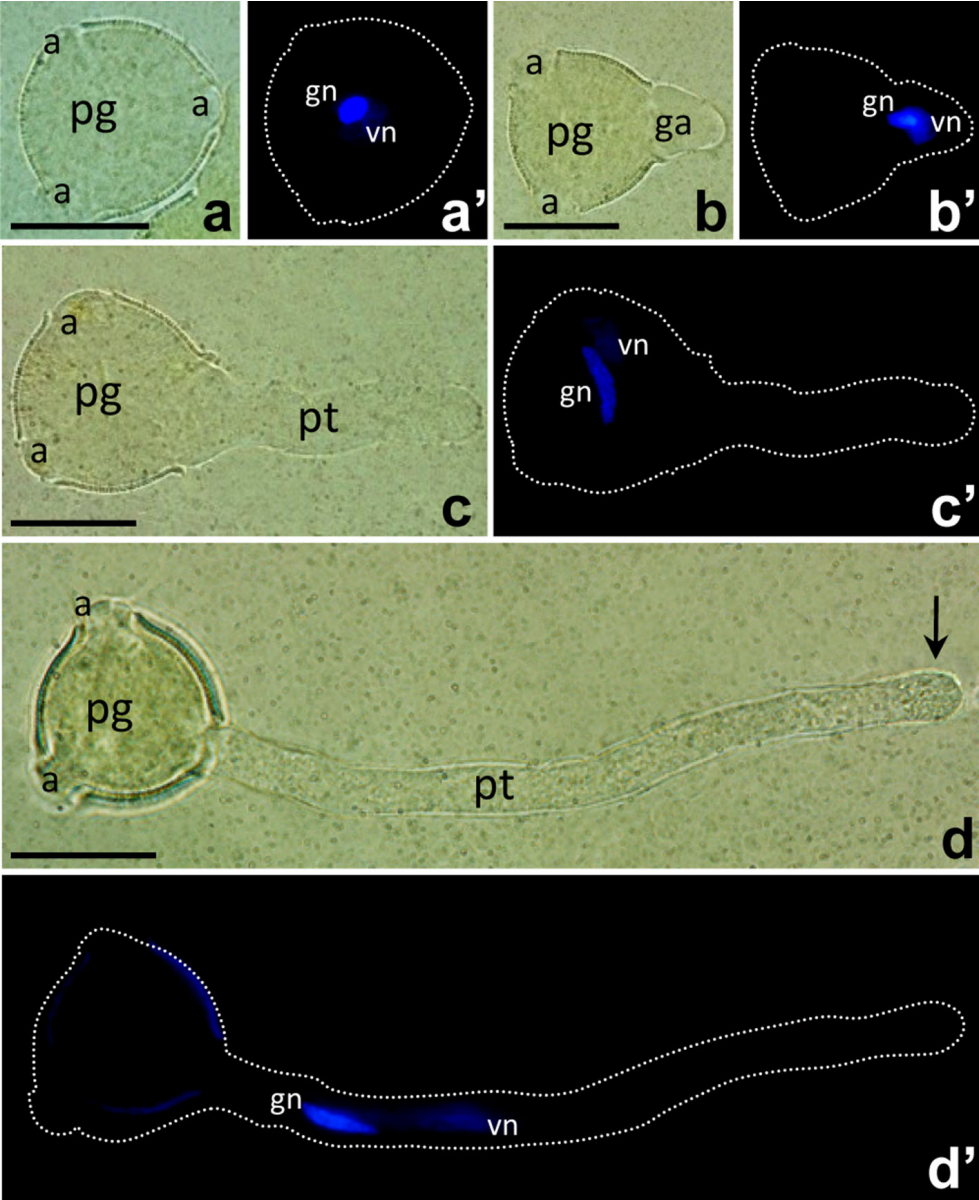
Morphology of *Petunia* germinating pollen (**a**, **b**) and growing pollen tubes (**c**, **d**); positions of vegetative and generative nuclei (*vn* and *gn*, respectively) in cultivated cells are visualized by DAPI staining (**a′**, **b′**, **c′**, **d′**). *Arrow* in **d** shows the pollen tube clear zone. *pg* pollen grain, *pt* pollen tube. *Bars* 50 μm

During in vitro hydration of mature *Petunia* dicellular pollen grain in the culture medium, three apertures begin to emerge (Fig. 1a), and two nuclei—the less-condensed vegetative nucleus and the highly condensed generative nucleus—are visible within the pollen grain cytoplasm by DAPI staining (Fig. 1a′). When the pollen tube starts to elongate (Fig. 1b), both nuclei (referred to as the MGU) move toward the germinal aperture (Fig. 1b′). However, in some cases, the MGU remains in the pollen grain region during pollen tube elongation in vitro (Fig. 1c, c′). After about two hours, pollen tubes reach a length equal to about four times the diameter of their pollen grains. In these long tubes, the vesicle-packed clear zone is evident at the apex (Fig. 1d, arrow), and the two nuclei can be observed to have moved through the tube toward its growing tip (Fig. 1d′). In vitro cultivated *Petunia* pollen tubes show a typical bidirectional “reverse fountain” cytoplasmic streaming pattern that is clearly visible by light microscopy (data not shown).

### Distribution of the *PhCRT* mRNA and CRT during *Petunia* pollen germination and pollen tube growth

**Fig. 2:**
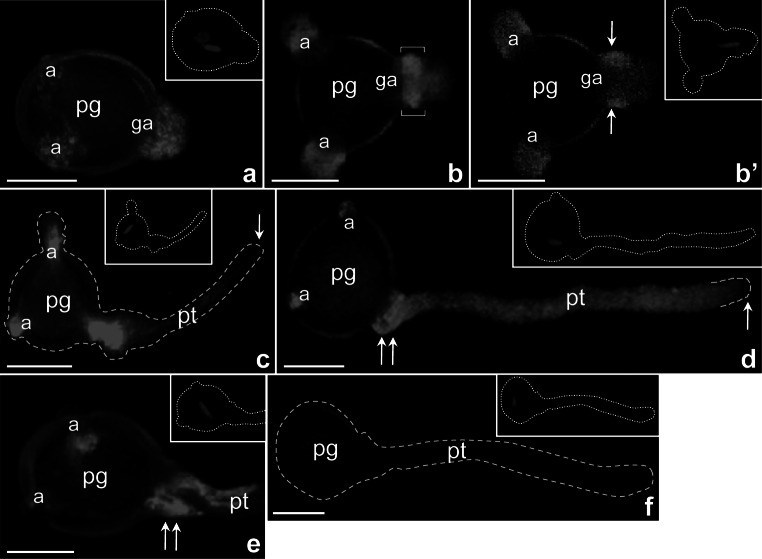
Localization of *PhCRT* mRNA in *Petunia* germinating pollen (**a**–**b′**) and growing pollen tubes (**c**–**e**). *Bracketed region* and *arrows* in **b**–**b′** show transcripts accumulation at the base of outgrowing tube, where they formed ring or collar in the cortical cytoplasm. *Arrows* in **c**, **d** show lack of fluorescence in the clear zone of growing pollen tubes (*pt*), and *double arrows* in **d**–**e** show specific localization of *PhCRT* mRNA at the base of elongated tubes. **f** negative control of FISH. *a* aperture, *ga* germinal aperture, *pg* pollen grain. *Bars* 50 μm

**Fig. 3:**
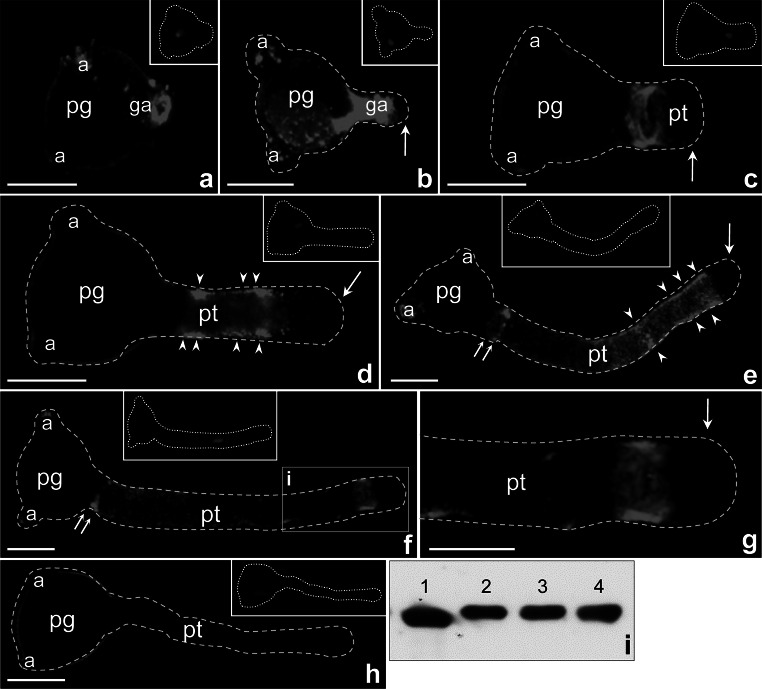
Localization of CRT protein in *Petunia* germinating pollen (**a**–**c**) and growing pollen tubes (**d**–**f**); **g** is a bigger magnification of marked region (**i**) in **f** showing the protein accumulation in the subapical zone of elongated pollen tube. *Arrows* in **b**–**e**, **g** show lack of fluorescence within the clear zone of growing pollen tubes (*pt*), *double arrows* in **e**, **f** show specific localization of CRT at the base of elongated tubes, and *arrow heads* in **d**–**e** show the signals localized on the peripheral cytoplasm of the growing tubes. **h** Negative control of immunolocalization. **i** Immunoblotting of crude protein extracts from *Petunia* anthers (*2*), dry pollen (*3*), pollen hydrated in culture medium (*4*), and maize anthers (*1*). *a* aperture, *ga* germinal aperture, *pg* pollen grain. *Bars* 50 μm

To determine the spatial distribution of CRT and its transcripts in in vitro germinating pollen and growing tubes, we separately performed FISH and immunostaining and imaged the samples by confocal microscopy. At the beginning of pollen germination, both *PhCRT* mRNA and CRT protein were detected predominantly at the aperture regions, and the strongest signals were always associated with the germinal aperture (Figs. 2a and 3a, respectively). As germination proceeded, the hybridization and immunolabeling signals were prominent in the cytoplasm of outgrowing pollen tubes (Figs. 2b and 3b). By performing optical sectioning, we observed that *PhCRT* transcripts accumulated at the base of outgrowing tubes, where they formed rings or collars in the cortical cytoplasm (bracketed region and arrows in Fig. 2b–b′). In very short pollen tubes, we observed the same pattern of CRT protein localization (Fig. 3c). Notably, immunofluorescence signals were never observed at the apex (Fig. 3b, c, arrows).

In elongated tubes of different lengths, *PhCRT* mRNA was diffusely distributed throughout the cytoplasm of the tube shank, extending from the base to the subapical zone (Fig. 2c, d). The clear zone of the pollen tube was always devoid of *PhCRT* mRNA (Fig. 2c, d, arrows). At the base of long tubes, *PhCRT* transcripts were abundant and formed a collar shape (Fig. 2d, e, double arrows). We observed similar, but not identical, patterns of CRT protein distribution in elongated pollen tubes. In the cytoplasm of shorter tubes, labeling was detected predominantly along the edge of the cell from the base to the subapical domain (Fig. 3d, arrowheads). In longer tubes, CRT preferentially localized to the distal regions (Fig. 3e), where intense labeling was observed adjacent to the peripheral cytoplasm of the tube (Fig. 3e, arrowheads). In elongated pollen tubes, the regions of the highest CRT localization were the subapical cytoplasm (Fig. 3f, g) and the cytoplasm at the base of the tubes (Fig. 3e, f, double arrows). As with *PhCRT* mRNA, the clear zone of pollen tubes was devoid of CRT labeling (Fig. 3b–e and g, arrows). We also observed that both *PhCRT* mRNA and CRT protein were associated with the inactive apertures of germinating pollen grains or growing pollen tubes (Figs. 2a–d and 3a, b, e, f).

We observed no labeling in controls, in which the *PhCRT* mRNA molecular probe or CRT PAb was omitted (Figs. 2f, 3h). The specificity of the CRT PAb was verified by immunoblotting of crude protein extracts from *Petunia* anthers, dry pollen, pollen hydrated in culture medium, and maize anthers used as a positive control (Fig. 3i). We observed a single band in every lane.

### Localization of *18S* rRNA and RER in *Petunia* germinating pollen and growing tubes

**Fig. 4:**
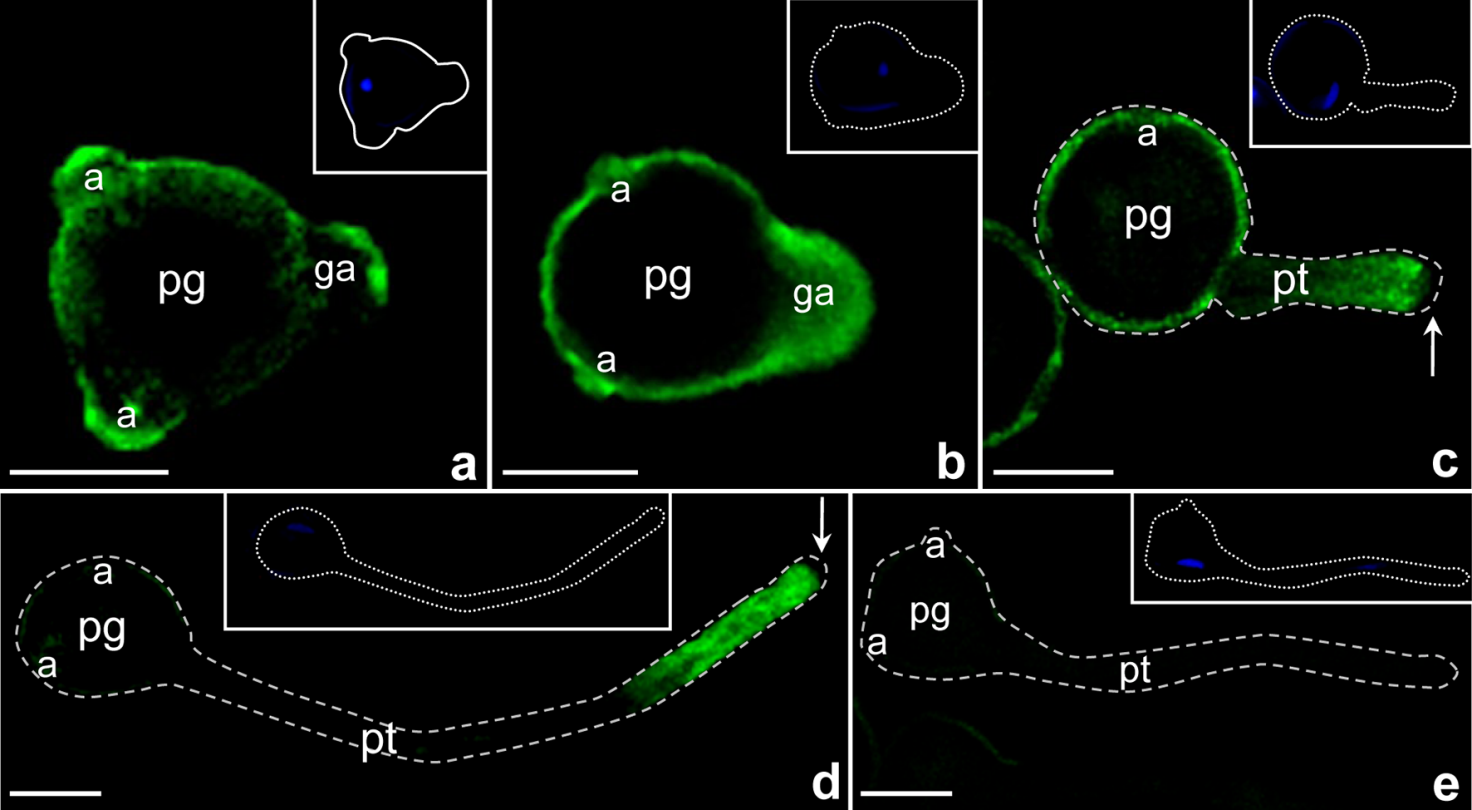
Distribution of *18S* rRNA in *Petunia* germinating pollen (**a**–**b**) and growing pollen tubes (**c**–**d**). *Arrows* in **c**, **d** show lack of hybridization signals within the clear zone of growing pollen tubes (*pt*).** e** Negative control of FISH. *a* aperture, *ga* germinal aperture, *pg* pollen grain. *Bars* 50 μm

The similar localization patterns of *PhCRT* mRNA and CRT protein in germinating pollen and growing tubes suggested that the sites of enrichment corresponded with sites of CRT translation. To address this possibility, we detected *Petunia 18S* rRNA by in situ hybridization. As *18S* rRNA is an essential component of the small ribosome subunit, the detected signal serves as a marker for enrichment of ribosomes. In pollen germinated in vitro, we observed *18S* rRNA labeling along the edge of the pollen grain cytoplasm, with the strongest signals associated with both the germinal and inactive apertures (Fig. 4a, b). As germination proceeded, *18S* rRNA preferentially accumulated in the cytoplasm of the outgrowing pollen tube (Fig. 4b). Although this signal was reminiscent of what we observed for both *PhCRT* mRNA and CRT protein, we did not observe any *18S* rRNA accumulation at the base of outgrowing tubes as was typical for CRT and its transcripts (compare Fig. 4b with Figs. 3c and 2b).

Longitudinal optical sections of elongated tubes showed that *18S* rRNA was predominantly localized in the distal, subapical cytoplasmic regions (Fig. 4c, d). The clear zone of growing pollen tubes was devoid of *18S* rRNA (arrows in Fig. 4c, d). This *18S* rRNA labeling pattern was generally similar to that observed for localization of CRT and its transcripts in elongated pollen tubes. However, we did not detect accumulation of *18S* rRNA at the base of short (Fig. 4c) or long (Fig. 4d) tubes. We also did not observe *18S* rRNA localization in the proximal tube shank or inactive apertures of elongated tubes (Fig. 4d). No labeling was observed when the probe was omitted (Fig. 4e).

Together, these results revealed several common sites of *PhCRT* mRNA, CRT protein, and *18S* rRNA localization in *Petunia* germinating pollen and growing tubes: aperture regions of germinated pollen/outgrowing tubes and cytoplasmic distal regions of elongated tubes. These data are summarized in Table 1.

**Table 1:** Common sites of *PhCRT* mRNA, CRT protein, and *18S* rRNA localization in *Petunia* germinating pollen, outgrowing pollen tubes, and elongated tubes

Molecule	Germinating pollen		Outgrowing pollen tube			Elongating pollen tube				
	*ga*	*a*	*ga*	*a*	*br*	*a*	*br*	*psh*	*dsh*	*saz*
*CRT* mRNA	+	±	+	±	+	+	+	+	+	+
CRT	+	±	+	±	+	+	+	±	+	+
*18S* rRNA	+	+	+	±	–	–	–	–	+	+

*a* inactive aperture of germinating pollen, *br* base region of growing tube, *dsh* distal shank region of the tube, *ga* germinal aperture of the pollen grain, *psh* proximal shank region of the tube, *saz* subapical zone of the pollen tube. Designations “+,” “±,” and “−” mean respectively “molecule always present,” “molecule sometimes present,” and “molecule not present”

**Fig. 5:**
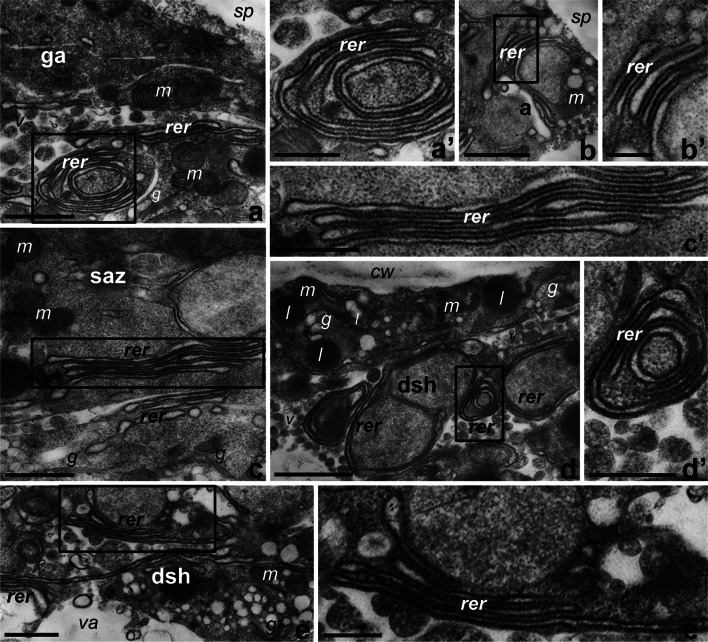
The presence of RER cisternae in the regions of *Petunia* germinating pollen (**a**–**b′**) and growing tubes (**c**–**e′**) in which accumulation of *PhCRT* mRNA, CRT protein, and *18S* rRNA was revealed. **a**–**a′** the germinal aperture (*ga*) of germinating pollen, **b**–**b′** inactive aperture (*a*) of germinating pollen, **c**–**c′** the subapical zone (*saz*) of the growing pollen tube, **d**–**d′** peripheral distal shank (*dsh*) of the pollen tube, **e**–**e′** central *dsh* of the pollen tube. **a′**, **b′**, **c′**, **d′**, and **e′** are the bigger magnifications of *marked regions* in respective photos. *g* Golgi stacks, *l* lipid body, *m* mitochondria, *rer* rough ER, *sp* sporoderm, *va* vacuole. *Bars* 1 μm (**a**–**e**), 500 nm (**a′**, **c′**, **e′**), and 250 nm (**b′**, **d′**)

The above results led us to propose that the common sites of *PhCRT* mRNA, CRT, and *18S* rRNA localization in germinating pollen and growing pollen tubes are enriched in ER membrane-bound ribosomes. To test this idea, we performed high-resolution electron microscopy on ultrathin longitudinal sections through in vitro cultivated cells. As shown in Fig. 5, we observed RER cisternae in the same regions in which we found accumulation of *PhCRT* mRNA, CRT protein, and *18S* rRNA: the germinal aperture (Fig. 5a, a′) and inactive apertures (Fig. 5b, b′) of germinated pollen; and the subapical zone (Fig. 5c, c′) and the peripheral (Fig. 5d, d′) and central (Fig. 5e, e′) regions of the distal shank of elongated tubes.

## Discussion

Together, our results show that the regions in which *PhCRT* mRNA, CRT protein, and *18S* rRNA are localized are also rich in RER. These sites include mainly the germinal aperture of germinating pollen and subapical regions of growing pollen tubes. This is indirect but clear evidence that *PhCRT* mRNA may be actively translated on the ER membrane-bound ribosomes in in vitro cultivated pollen and pollen tubes.

It is generally accepted that protein synthesis is compartmentalized in eukaryotic cells and that newly synthesized mRNAs are partitioned between the cytosol and ER (see review by Cui and Palazzo 2014). Soluble cytosolic and nucleoplasmic proteins are synthesized on free ribosomes, whereas mRNAs encoding topogenic signal-bearing proteins such as secretory, organellar, and integral membrane proteins are translated on ER-bound polysomes. The accepted model to explain how mRNAs are partitioned to the ER is that mRNAs encoding ER-lumenal, secretory, or transmembrane proteins initiate translation on free cytosolic ribosomes. These classes of proteins contain an N-terminal ER signal sequence or a transmembrane segment that emerges from the ribosome and is recognized by the signal recognition particle (SRP) in the cytoplasm. SRP directs the mRNA–ribosome complex to the SRP receptor/protein translocator localized within the ER membrane. Several soluble ER-lumenal proteins contain also a specific C-terminal sequence (KDEL/HDEL) that prevents their secretion to other cell compartments or outside the cell (see review by Cui and Palazzo 2014). As an ER lumen-resident protein, CRT has both an N-terminal ER-targeting sequence and a KDEL/HDEL retention signal (see reviews by Jia et al. 2009; Michalak et al. 2009). Our previous data clearly showed that the *PhCRT* transcript identified in *Petunia* pollen/pollen tubes encodes a protein that contains both an N-terminal ER signal sequence and an HDEL motif (Lenartowski et al. 2014). Combining these above facts with our results here, we conclude that CRT is translated on ER membrane-bound ribosomes during pollen germination and pollen tube growth.

Although the canonical model of mRNAs partitioning between the cytosol and ER is well established, evidence for ribosome-independent mRNA association with the ER, as well as signal sequence- and translation-independent mRNA localization to the ER, has been reported (Phytila et al. 2008; Chen et al. 2011). It has been hypothesized that specific proteins can bind directly to mRNA and promote ER anchoring of certain transcripts (see review by Cui and Palazzo 2014). Three pieces of evidence suggest that this alternative pathway occurs during *CRT* mRNA translation on ER-bound polysomes in germinating and elongating pollen tubes. First, Phytila et al. 2008 showed that *CRT* transcripts localized to the ER membrane in cells with little to no SRP. Second, Cui et al. (2012) demonstrated that *CRT* mRNAs can associate with the ER independently of ribosomes and translation and those membrane-bound receptors are probably required for maintenance of these transcripts on the ER surface. One of the best candidates for anchoring *CRT* mRNA to the ER is ER membrane-bound protein p180, which contains a region that binds directly to RNA. Third, some mRNAs that encode ER-resident proteins appear more tightly bound to the ER than others, suggesting that different targeting mechanisms act synergistically to enhance ER anchoring of certain transcripts (Chen et al. 2011; Cui et al. 2012). Therefore, we suggest that in germinating pollen and growing tubes, CRT may be translated on the ER membrane-bound ribosomes in both SRP-dependent and SRP-independent manners. However, further investigations are needed to confirm this hypothesis.

Our current studies have revealed that the main sites of *CRT* mRNA, CRT protein, *18S* rRNA, and RER cisternae co-existence are the germinal aperture of the pollen grain and the subapical, cytoplasmic regions of elongating pollen tubes. Although RER is highly abundant starting behind the inverted cone of Golgi-derived secretory vesicles and throughout the rest of the tube cytoplasm (see review by Cheung and Wu 2007), it appears that CRT translation on membrane-bound ribosomes is restricted to the subapical cytoplasm of elongated tubes. Selective targeting of plant *CRT* mRNA to specific ER subdomains to promote local synthesis and enrichment of CRT protein has also been reported in maize callus cells (Šamaj et al. 2008). In growing pollen tubes, ER moves distally along actin tracks in the cortical region of the shank, reverses its direction at the sub-apex in the cortical actin fringe region, and then moves proximally through the central core of the tube shank (Lovy-Wheeler et al. 2005). We have detected both CRT and its transcripts at the base of the elongating tube, in the shank region (both peripherally and centrally), and in the subapical domain. Such distributions of *CRT* mRNA and CRT may indicate that these molecules are transported to the apical domain via the bidirectional “reverse fountain” cytoplasmic streaming that occurs in growing pollen tubes. Moreover, the strong CRT enrichment adjacent to the cell wall of the tube may represent cortical-associated ER rich in CRT.

We currently do not know the function of CRT and its transcripts that are localized at the base of growing pollen tubes or in the inactive apertures. The molecular mechanism of the “reverse fountain” streaming activity at the base of the tube is unknown, but perhaps CRT, through its ability to regulate Ca^2+^ homeostasis, modulates the activity of certain actin-binding proteins and thereby regulates actin organization. The presence of *CRT* mRNA and CRT protein in the inactive apertures may be an in vitro phenomenon because we did not observe such localization in planta (Lenartowski et al. 2014, 2015). Unlike most plant cells, which dedifferentiate and lose polarity upon in vitro culture, cultured pollen maintains its polarity and developmental identity (see review by Qin and Yang 2011). In fact, in vitro pollen tubes grow synchronously and uniformly, and exhibit a highly polarized cytoplasmic organization in which the apical region is packed with vesicles. However, pollen tube adhesion and directional extension were found only in pollen tubes grown in vivo. Therefore, we suspect that in the absence of pistil guidance mechanisms, some proteins (such as CRT) can be abundant in inactive apertures without negative consequences on pollen tube tip growth. The presence of CRT and its transcripts in the inactive apertures may rather reflect endogenous stores of mature pollen than active translation during pollen tube growth, since it has long been known that mature pollen grains contain many different mRNAs (named as long-lived mRNAs) and proteins.

We found that in both elongated and highly extended pollen tubes, CRT was enriched in the subapical cytoplasm; this localization corresponds to localization of ER and Golgi stacks. These organelles were seen to be most concentrated at ~5–25 μm from the tube apex and outside the inverted cone of vesicles (see review by Cheung and Wu 2007). Similar intense CRT labeling at the borders of the subapical zone has also been reported in pollen tubes growing in vitro (Nardi et al. 2006) and in situ (Lenartowska et al. 2002, 2009; Lenartowski et al. 2015). Nardi et al. (2006) speculated that CRT is involved in reversing the cytoplasmic streaming at the tip. It has long been known that pollen tubes possess a tip-focused Ca^2+^ gradient that is essential for pollen germination and pollen tube growth. The cytoplasmic Ca^2+^ reaches 2–10 μM in the apex and drops dramatically to a basal level of 20–200 nM within 20 μm of the subapical region (see reviews by Hepler et al. 2011, 2013; Steinhorst and Kudla 2013). We thus propose that, in the subapical zone of growing pollen tubes, CRT in the abundant ER and Golgi compartments serves to sequester Ca^2+^ in releasable intracellular stores (Lenartowska et al. 2002, 2009; Nardi et al. 2006; Lenartowski et al. 2015, and present paper). The dissipation of Ca^2+^ behind the tube apex is thought to be regulated by Ca^2+^-ATPases, which are likely located on the ER close to the apical region of the pollen tube (see review by Sze et al. 2006). Therefore, CRT translated on ER membrane-bound ribosomes in the regions where its activity is required for stabilization of tip-focused Ca^2+^ gradient in growing pollen tubes seems to be a key regulatory element of bidirectional “reverse fountain” cytoplasmic streaming.

### **Author contribution statement**

AS, RL, and ML conceived and designed the experiments; AS, RL, and DJS performed the experiments; AS, RL, and ML analyzed the data; and RL and ML wrote the paper.

## Abbreviations

Ca^2+^: Calcium/calcium ions
*CRT*/CRT: Calreticulin gene/protein
CRT PAb: Polyclonal antibody against calreticulin
ER: Endoplasmic reticulum
MGU: Male germ unit
RER: Rough endoplasmic reticulum
*PhCRT*: *Petunia hybrida* calreticulin gene
SRP: Signal recognition particle
